# Ethylene glycol and glycolic acid production from xylonic acid by *Enterobacter cloacae*

**DOI:** 10.1186/s12934-020-01347-8

**Published:** 2020-04-15

**Authors:** Zhongxi Zhang, Yang Yang, Yike Wang, Jinjie Gu, Xiyang Lu, Xianyan Liao, Jiping Shi, Chul Ho Kim, Gary Lye, Frank Baganz, Jian Hao

**Affiliations:** 1 School of Life Science, Shanghai University, Shanghai, 200444 People’s Republic of China; 2 Lab of Biorefinery, Shanghai Advanced Research Institute, Chinese Academy of Sciences, Shanghai, 201210 People’s Republic of China; 3 Department of Biochemical Engineering, University College London, Gordon Street, London, WC1H 0AH UK; 4 Microbial Biotechnology Research Center, Jeonbuk Branch Institute, KRIBB, Jeongeup, Jeonbuk 556212 South Korea; 5 University of Chinese Academy of Sciences, Beijing, 100049 People’s Republic of China; 6 School of Life Science and Technology, ShanghaiTech University, Shanghai, People’s Republic of China

**Keywords:** *Enterobacter cloacae*, Ethylene glycol, Glycolic acid, Xylonic acid, Xylose

## Abstract

**Background:**

Biological routes for ethylene glycol production have been developed in recent years by constructing the synthesis pathways in different microorganisms. However, no microorganisms have been reported yet to produce ethylene glycol naturally.

**Results:**

Xylonic acid utilizing microorganisms were screened from natural environments, and an *Enterobacter cloacae* strain was isolated. The major metabolites of this strain were ethylene glycol and glycolic acid. However, the metabolites were switched to 2,3-butanediol, acetoin or acetic acid when this strain was cultured with other carbon sources. The metabolic pathway of ethylene glycol synthesis from xylonic acid in this bacterium was identified. Xylonic acid was converted to 2-dehydro-3-deoxy-d-pentonate catalyzed by d-xylonic acid dehydratase. 2-Dehydro-3-deoxy-d-pentonate was converted to form pyruvate and glycolaldehyde, and this reaction was catalyzed by an aldolase. d-Xylonic acid dehydratase and 2-dehydro-3-deoxy-d-pentonate aldolase were encoded by *yjhG* and *yjhH*, respectively. The two genes are part of the same operon and are located adjacent on the chromosome. Besides *yjhG* and *yjhH*, this operon contains four other genes. However, individually inactivation of these four genes had no effect on either ethylene glycol or glycolic acid production; both formed from glycolaldehyde. YqhD exhibits ethylene glycol dehydrogenase activity in vitro. However, a low level of ethylene glycol was still synthesized by *E. cloacae* Δ*yqhD*. Fermentation parameters for ethylene glycol and glycolic acid production by the *E. cloacae* strain were optimized, and aerobic cultivation at neutral pH were found to be optimal. In fed batch culture, 34 g/L of ethylene glycol and 13 g/L of glycolic acid were produced in 46 h, with a total conversion ratio of 0.99 mol/mol xylonic acid.

**Conclusions:**

A novel route of xylose biorefinery via xylonic acid as an intermediate has been established.

## Background

Ethylene glycol is an important bulk chemical that is used primarily as a precursor for polyethylene terephthalate, polyurethane, and polyethylene succinate synthesis. Ethylene glycol is also used as feed stock for the synthesis of glyoxal, glycolic acid, methyl glycolate and other chemicals [1]. Industrially, ethylene glycol is produced chemically from ethylene. However, with the development of synthetic biology, ethylene glycol production by biological routes has become a research hotspot in recent years. Liu et al. constructed an ethylene glycol synthesis pathway in *Escherichia coli*. This pathway consists of four steps: xylose → xylonate → 2-dehydro-3-deoxy-d-pentonate → glycolaldehyde → ethylene glycol. The first step converting xylose to xylonic acid was catalyzed by d-xylose dehydrogenase, which was originally obtained from *Caulobacter crescentus*. The residual three steps were catalyzed by host native enzymes of d-xylonic acid dehydratase, 2-dehydro-3-deoxy-d-pentonate aldolase, and aldehyde reductase, respectively. This strain produced 11.7 g/L ethylene glycol from 40 g/L xylose and glycolic acid as a by-product of this process [2]. Beside this pathway, a synthetic pathway of xylose → xylulose → xylulose-1P → glycolaldehyde → ethylene glycol was constructed in *E. coli* to produce ethylene glycol from xylose [3]. Following these strategies, other pentoses were used as substrates for ethylene glycol synthesis in *E. coli* [4]. Beside pentose, glucose was also used for ethylene glycol production. This synthesis pathway was constructed in *Corynebacterium glutamicum* and *E. coli* by using serine as an intermediate [5, 6].

Xylose is the second most abundant sugar in nature after glucose. Xylose can be used as a carbon source for culture of microorganisms. However, the catabolism of xylose by microorganisms is not as easy as that of glucose. In our previous research, xylonic acid production by *Klebsiella pneumoniae* was developed, and this process has a high conversion ratio and productivity [7]. Thus we proposed to use xylonic acid as an intermediate for xylose biorefinery. The enzymes that catalyze the conversion of xylose to xylonic acid belong to three classes based on the cofactor used. Glucose dehydrogenase was identified to catalyze the reaction in *K. pneumoniae*, and this enzyme is located in the inner membrane of the periplasmic space and uses pyrroloquinoline quinine (PQQ) as the cofactor. D-xylose dehydrogenase from *Trichoderma reesei* uses NADPH as the cofactor [8] whereas d-xylose dehydrogenase from *C. crescentus* uses NADH as the cofactor [9]. These two D-xylose dehydrogenases are located in the cytoplasm. Different cofactors are used and the different location of the enzymes lead to the different efficiency of xylonic acid production. 103 g/L xylonic acid was produced in 79 h by *K. pneumoniae* using glucose dehydrogenase [7]. While only 19 g/L xylonic acid was produced in 150 h of culture by *Trichoderma reesei*, using a NADPH dependent d-xylose dehydrogenase [8]. 39 g/L xylonic acid was produced after 36 h of culture by *E. coli*, using a NADH dependent d-xylose dehydrogenase [9].

**Fig. 1:**
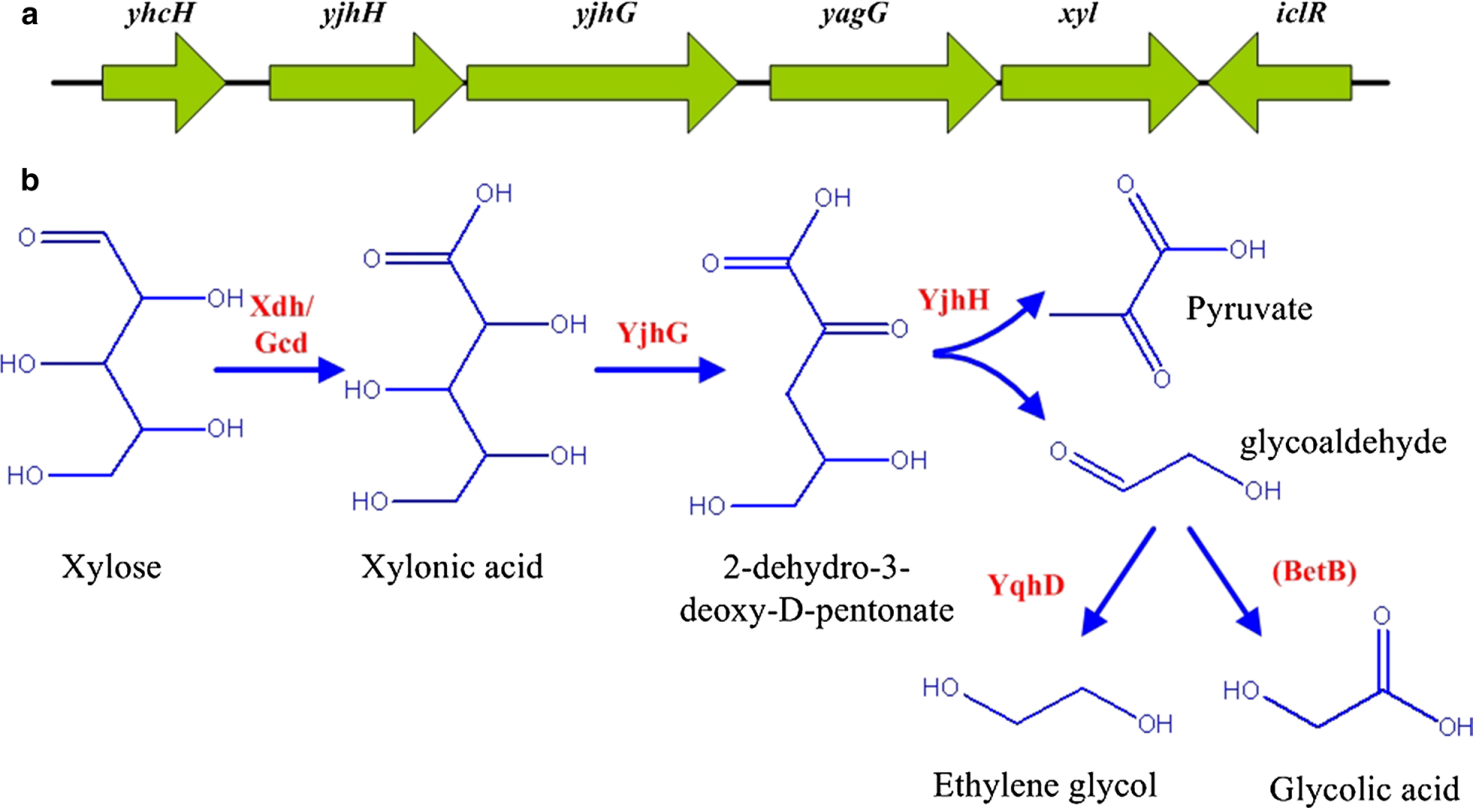
Ethylene glycol and glycolic acid synthesis pathway and *yjh* operon of *E. cloacae.***a***yjh* operon of *E. cloacae* containing 6 genes. **b** Metabolic pathway of ethylene glycol and glycolic acid synthesis from xylose. Xdh: d-xylose dehydrogenase of *C. crescentus*; GcD glucose dehydrogenase of *K. pneumoniae*; YjhG 2-dehydro-3-deoxy-d-pentonate aldolase of *E. cloacae*; YjhH: aldehyde reductase of *E. cloacae*; YqhD: alcohol dehydrogenase of *E. cloacae*; BetB: aldehyde dehydrogenase of *E. cloacae*

Unlike gluconic acid, which is an intermediate of the glucose oxidization pathway [10], xylonic acid cannot be further catabolized by *K. pneumoniae*. Therefore, in this work xylonic acid utilizing microorganisms were screened from nature, and an *Enterobacter cloacae* strain was selected. This bacterium was a native ethylene glycol producer, and the metabolic pathway of ethylene glycol and glycolic acid synthesis from xylonic acid was identified (Fig. 1b). Furthermore, the process conditions for ethylene glycol and glycolic acid production were optimized.

## Results

### Screening of xylonic acid utilizing microorganisms

Xylonic acid utilizing microorganisms were enriched from soil samples and 4 colonies with different morphologies were isolated from LB agar plates and cultured in flasks. *E. coli* W3110 was also cultured at the same time as a control. Fermentation results of these strains are shown in Table 1.

**Table 1:** Metabolites produced by xylonic acid utilizing microorganisms tested

Strains	Residual xylonic acid (g/L)	Metabolites (g/L)		
		Ethylene glycol	Glycolic acid	Acetic acid
1	0	11.1	3.1	0
2	27.5	0	0	0
3	28.0	0	0	0
4	37.9	0	0	0
W3110	10.1	3.3	1.9	2.5

Xylonic acid was consumed by isolated strains (1–3) and *E. coli* W3110, but not by strain 4. Of the xylonic acid utilizing strains, no known metabolites were detected in the broth of strains 2 and 3. For strain 1 and *E. coli* W3110, ethylene glycol (assumed) and glycolic acid (assumed) were the major metabolites. The identification of ethylene glycol and glycolic acid are shown in the following section. Acetic acid was found as a metabolite of *E. coli* W3110, but not for any of the other strains.

Strain 1 has a higher ethylene glycol and glycolic acid productivity and yields than *E. coli* W3110. This strain was selected for further investigation. The 16S rRNA gene of this strain was sequenced and has been submitted to GenBank with the accession number of MG779638. The dendrogram of strain 1 and some related strains are shown in Additional file 1: Figure S1. Based on 16S rRNA gene sequence and the dendrogram, strain 1 was tentatively identified as *E. cloacae*, and named *E. cloacae* S1. The genome of this strain was subsequently sequenced and the raw sequence has been submitted to GenBank with the accession number of VSZU00000000. This strain was used for further investigation.

### Ethylene glycol and glycolic acid identification

^1^H and ^13^C NMR spectral data of the presumed glycolic acid sample compared to the spectra of a standard glycolic acid (Sodium salt commercial product) are given in Additional file 1: Figure S2A, B. ^1^H NMR chemical shift for CH_2_ of glycolic acid was 3.83 and 4.00 ppm for sample and standard, respectively. ^13^C NMR chemical shifts of glycolic acid were 179.52 (C1), 60.95 (C2) ppm for the sample, and 176.14 (C1), 59.12 (C2) ppm for the standard. The NMR data of the sample correlated well with the standard glycolic acid data. From this comparison, it was concluded that the compound was glycolic acid.

HPLC chromatograms and GC chromatograms of ethylene glycol are given in Additional file 1: Figure S2C, D. The retention times of standard ethylene glycol and sample were both 12.2 min for HPLC and both 8.2 min for GC analysis. These results confirmed that ethylene glycol was the presumed metabolite.

### Carbon sources utilization ability of *E. cloacae* S1

To determine the range of carbon sources that can be utilised by *E. cloacae* S1 the strain was cultured in flasks with M9 medium using either xylonic acid, xylose, glucose, gluconic acid, 2-ketogluconic acid or glycerol as the sole carbon source and the metabolites detected are listed in Additional file 1: Table S1. Ethylene glycol and glycolic acid were the main metabolites of *E. cloacae* S1 using xylonic acid as the sole carbon source. However, the two chemicals were not synthesized by this strain using any of the other carbon sources tested. 2,3-Butanediol and acetic acid were the major metabolites using xylose and 2-ketogluconic acid as the sole carbon sources, respectively. Acetoin and 2,3-butanediol were the major metabolites using glycerol as the sole carbon. When using glucose or gluconic acid as the sole carbon source, acetic acid, acetoin, and 2,3-butanediol were all synthesized by this strain.

### Gene recombination method development

Red recombinase assisted gene replacement of *E. cloacae* was developed as shown in the Method section based on the method we developed in *K. pneumoniae* [11]. pIJ790 is a plasmid that contains the red recombinase genes and used in *E. coli* for gene recombination [12]. However, this plasmid could not be used directly for gene recombination in *E. cloacae.* pSARI is a low copy number plasmid containing a temperature-inducible promoter and kanamycin resistance gene. pSARI can be transferred into *E. cloacae* and was used for red recombinase meditated gene manipulations. Gene recombination using linear DNA with 39 and 40 nt homologous extensions that was directly amplified from plasmid pIJ778 was tried first. However, no colonies were obtained on selection plates. So linear DNA with 500 bp of homologous regions was used for gene recombination in *E. cloacae*. Commonly, 100 colonies were obtained in a single recombination experiment using this method.

### Identification of genes responsible for glycolaldehyde synthesis from xylonic acid

**Fig. 2:**
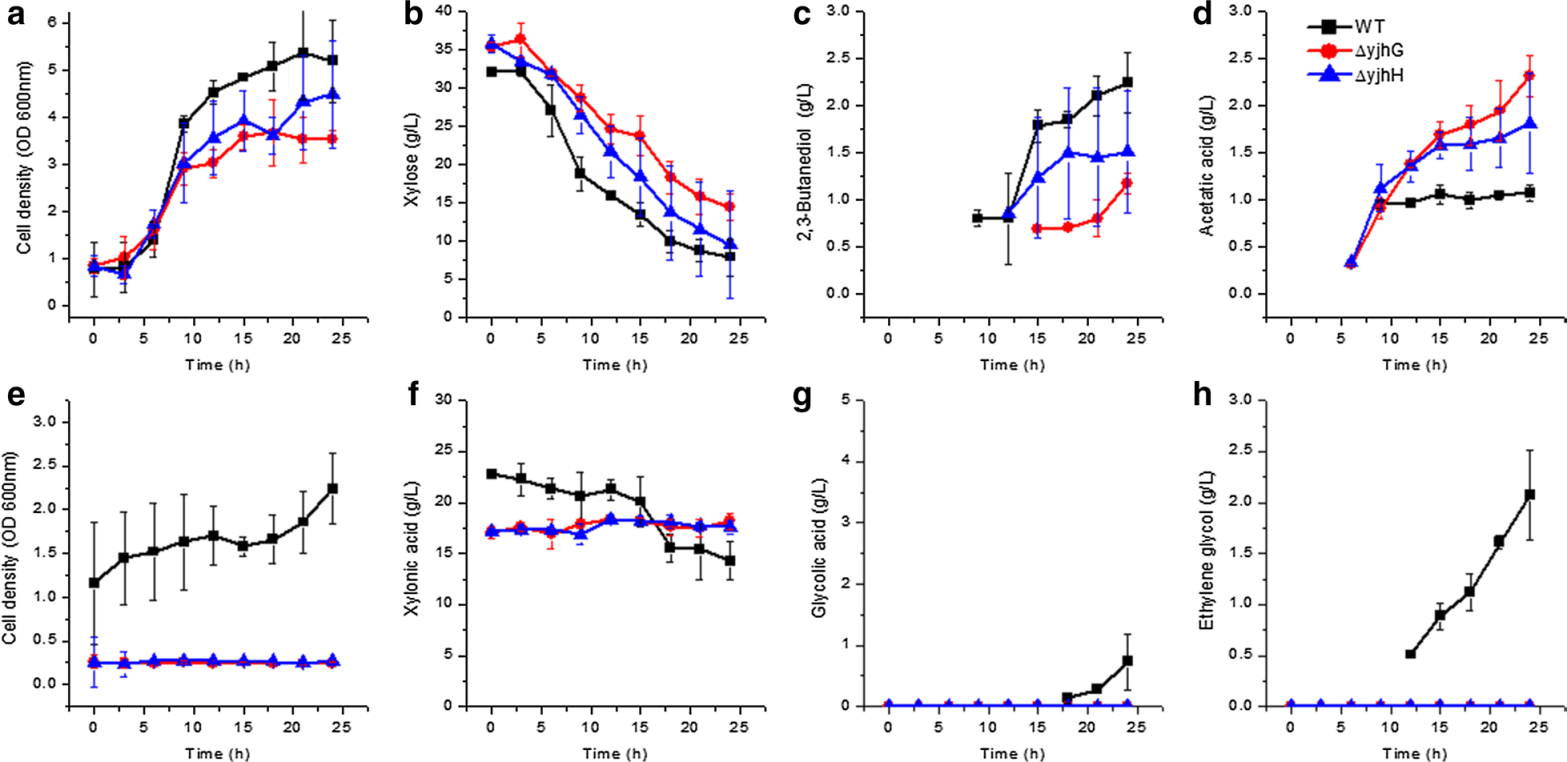
Growth and metabolite production of *E. cloacae* Δ*yjhG* and *E. cloacae* Δ*yjhH* grown on xylose (**a**–**d**) and xylonic acid (**e**–**h**) in shake flask batch culture. **a**–**d** Cell density, xylose utilization, 2,3-butanediol, and acetic acid production. **e**–**h** Cell density, xylonic acid utilization, glycolic acid, and ethylene glycol production. WT: *E. cloacae* S1 (filled square), ΔyjhG: *E. cloacae* Δ*yjhG* (filled circle); ΔyjhH: *E. cloacae* Δ*yjhH* (filled triangle). Data points are the average of n = 3; error bars represent standard error

There are two d-xylonic acid dehydratases (YjhG, YagF) catalyzing the conversion of xylonic acid to 2-dehydro-3-deoxy-d-pentonate, and two 2-dehydro-3-deoxy-d-pentonate aldolases (YjhH, YagE) that catalyze the conversion of 2-dehydro-3-deoxy-d-pentonate to glycolaldehyde in *E. coli* [2]. *yjhG*, *yagF*, *yjhH* and *yagE* of *E. coli* were blasted against the NCBI database and the genome of *E. cloacae* S1 to find the homologous genes of *E. cloacae*. However, only homologues of *yjhG* (93% identities) and *yjhH* (93% identities) were found. The two genes were located nearby in the *yjh* operon (Fig. 1a). Beside, this operon contains genes of *yhcH, yagG, xyl* and *iclR*, which encoding a beta subunit of beta-galactosidase, a sugar transporter, a beta-d-xylosidase, and a regulatory gene, respectively. *yjhG* and *yjhH* were knocked out individually to generate mutant strains of *E. cloacae* Δ*yjhG* and *E. cloacae* Δ*yjhH*, respectively. Physiological characteristics of these strains were determined by culturing them in M9 medium with xylonic acid or xylose as the sole carbon source, and results are presented in Fig. 2.

Growing with xylose as the sole carbon source 2.2, 1.2 and 1.5 g/L of 2,3-butanediol and 1.1, 2.3 and 1.8 g/L acetic acid were synthesized after 24 h culture by *E. cloacae* S1, *E. cloacae* Δ*yjhG* and *E. cloacae* Δ*yjhH*, respectively. There was not any distinct differences between these strains for xylose utilization. Using xylonic acid as the sole carbon source, 2.1 g/L ethylene glycol and 0.7 g/L glycolic acid were synthesized by *E. cloacae* S1. However, *E. cloacae* Δ*yjhG* and *E. cloacae* Δ*yjhH* were unable to grow in this medium, and no metabolites were synthesized.

### The roles of other genes in the *yjh* operon on xylose and xylonic acid catabolism

**Fig. 3:**
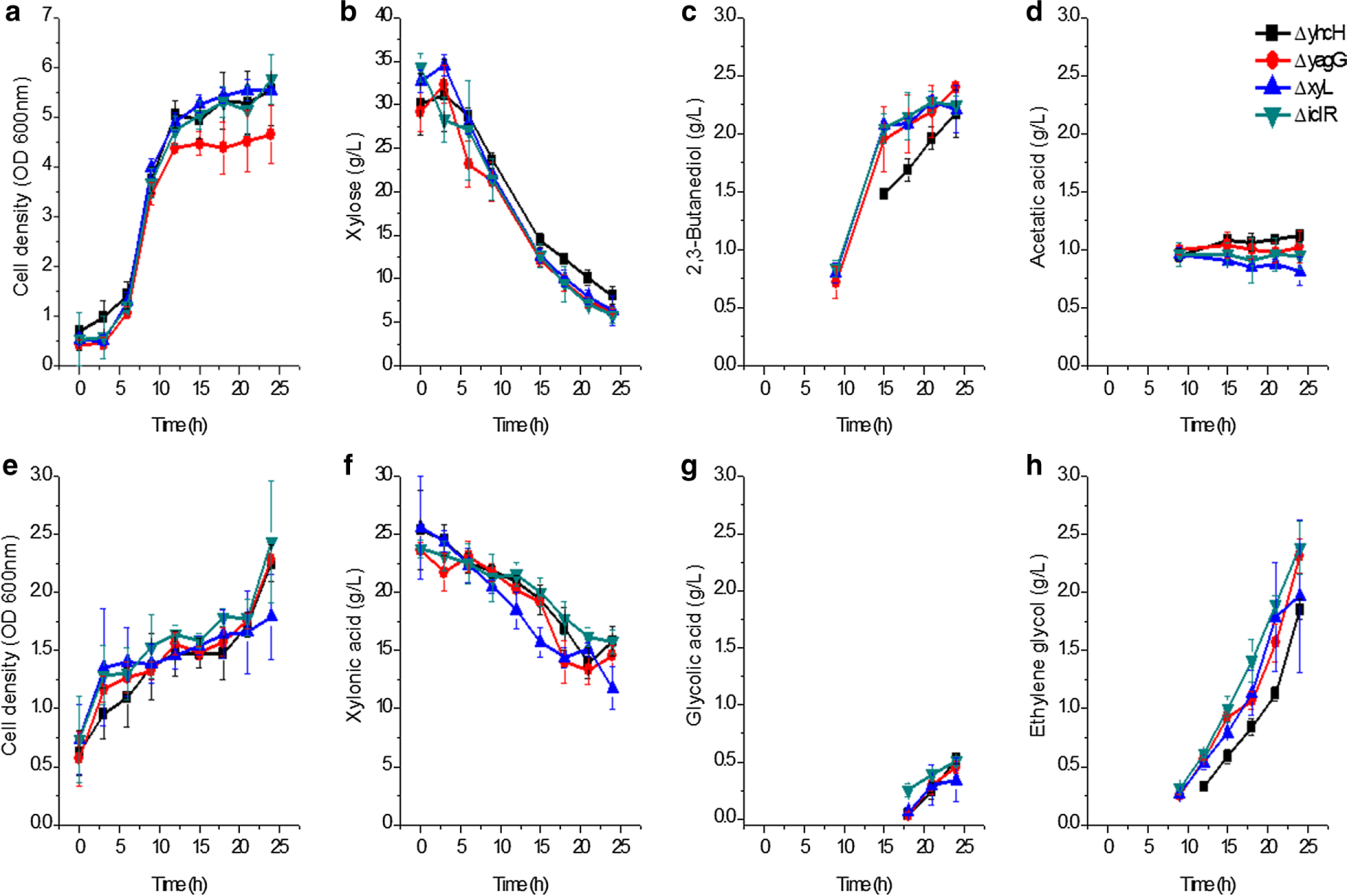
Growth and metabolite production of *E. cloacae* Δ*iclR, E. cloacae* Δ*yhcH, E. cloacae* Δ*yagG and E. cloacae* Δ*xyL* grown on xylose (**a**–**d**) and xylonic acid (**e**–**h**) in shake flask batch culture. **a**–**d** Cells growth, xylose utilization, 2,3-butanediol, and acetic acid production; **e**–**h** Cells growth, xylonic acid utilization, glycolic acid, and ethylene glycol production. ΔyhcH: *E. cloacae* Δ*ychH* (filled square), ΔyagG: *E. cloacae* Δ*yagG* (filled circle); ΔxyL: *E. cloacae* Δ*xyL* (filled up triangle) ΔiclR: *E. cloacae* Δ*iclR* (filled down triangle). Data points are the average of n = 3; error bars represent standard error

As *yjhG* and *yjhH* are responsible for xylonic acid catabolism it was suspected that other genes in the same operon might also be related to xylose or xylonic acid catabolism. *iclR*, *yhcH*, *yagG*, and *xyL* were disrupted individually to obtain strains *E. cloacae* Δ*iclR, E. cloacae* Δ*yhcH, E. cloacae* Δ*yagG* and *E. cloacae* Δ*xyL*, respectively. Physiological characteristics of these four strains were determined, and the results are presented in Fig. 3.

Xylose was used by *E. cloacae* Δ*iclR*, *E. cloacae* Δ*yhcH*, *E. cloacae* Δ*yagG*, and *E. cloacae* Δ*xyL*, and 2.2–2.3 g/L of 2,3-butanediol were produced by these strains. The cell growth and 2,3-butanediol synthesis were comparable to that of *E. cloacae* S1 (shown in Fig. 2). Xylonic acid was used by all these strains, and 0.3–0.5 g/L of glycolic acid and 1.9–2.4 g/L of ethylene glycol were synthesized by these strains. Also, these titers were similar to that of *E. cloacae* S1 (shown in Fig. 2). On the whole, the fermentation results showed that there were no distinct differences between the wild type strain and these mutants regarding xylose and xylonic acid utilization.

### Identification of genes responsible for ethylene glycol synthesis from glycolaldehyde

YqhD, a NADPH-dependent aldehyde reductase, was shown to catalyze the conversion of glycolaldehyde to ethylene glycol in *E. coli* [2]. Homologous gene of *yqhD* was amplified from *E. cloacae* S1. *yqhD* of *E. cloacae* S1 was 81% identical to that of *E. coli* W3110 suggesting that it also uses NADPH as cofactor. The ethylene glycol dehydrogenase activity of purified YqhD and the cell lysate of *E. cloacae* S1 were assayed using either NADH or NADPH as cofactor.

Ethylene glycol dehydrogenase activities of cell lysate of *E. cloacae* S1 using NADH or NADPH as cofactor were 0.006 ± 0.003 and 0.13 ± 0.005 U/mgP, respectively. Whereas the activity of purified YqhD was 0.004 ± 0.0005 and 0.175 ± 0.003 U/mgP of that using NADH or NADPH as the cofactor respectively. These results confirmed that the ethylene glycol dehydrogenase in *E. cloacae* S1 uses NADPH as the cofactor, and YqhD of *E. cloacae* S1 is an ethylene glycol dehydrogenase.

**Fig. 4:**
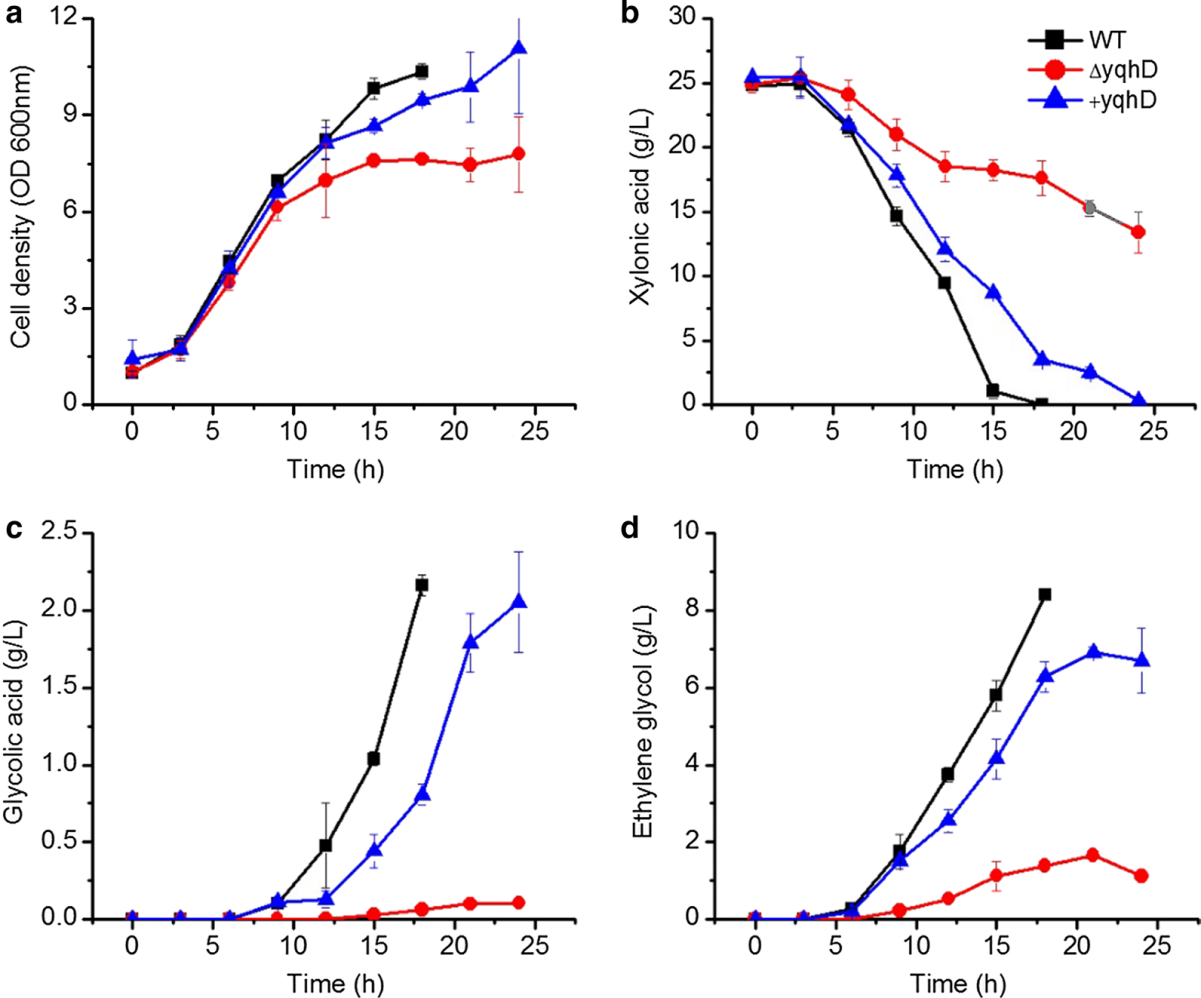
Growth and metabolite production of *E. cloacae* S1, *E. cloacae* Δ*yqhD* and *E. cloacae *+* yqhD* grown on xylonic acid in shake flask batch culture. *E. cloacae* S1 (filled square), *E. cloacae* Δ*yqhD* (filled circle) and *E. cloacae *+ *yqhD* (filled triangle). Data points are the average of n = 3; error bars represent standard error

To further investigate the in vivo function of *yqhD* in ethylene glycol formation, *yqhD* was knocked out and an YqhD over-expressing strain was constructed. *E. cloacae* S1, *E. cloacae* Δ*yqhD* and *E. cloacae *+ *yqhD* were cultured in flasks for ethylene glycol production. Fermentation medium was used, and the results are presented in Fig. 4.

Xylonic acid was exhausted by *E. cloacae* S1 after 18 h of culture, and 8.3 g/L ethylene glycol and 2.1 g/L of glycolic acid were produced. Xylonic acid utilization by *E. cloacae* Δ*yqhD* was much slower, however, ethylene glycol synthesis ability was not totally lost; the strain still produced 1.6 g/L of ethylene glycol. Similar to ethylene glycol, glycolic acid synthesized by *E. cloacae* Δ*yqhD* was decreased to 0.1 g/L. The final levels of ethylene glycol and glycolic acid produced by *E. cloacae *+ *yqhD* were only slightly lower compared to that of the wild-type strain. These results indicate YqhD is responsible for the conversion of glycolaldehyde to ethylene glycol in vivo. However, other ethylene glycol dehydrogenase isoenzymes exist in the cell that could explain the small quantities of ethylene glycol synthesized by the deletion mutant.

### Identification of genes responsible for glycolic acid synthesis from glycolaldehyde

*aldA* encoding an aldehyde dehydrogenase that is responsible for glycolic acid synthesis from glycolaldehyde in *E. coli* [2]. However, no homologous genes of *aldA* were found in the genome of *E. cloacae* S1. *aldB*, *betB*, *ad1*, and *ad2* that are presumed to be aldehyde dehydrogenases or putative aldehyde dehydrogenases in the genome of *E. cloacae* were cloned and over-expressed in *E. coli* to obtain *E. coli* BL21/aldB, *E. coli* BL21/betB, *E. coli* BL21/ad1, and *E. coli* BL21/ad2. Purified enzymes of these gene products were obtained from the lysate of these strains and analyzed for their glycolaldehyde dehydrogenase activities. The cell lysate of *E. cloacae* S1 was used as a control for the glycolaldehyde dehydrogenase activity assay. The results are shown in Additional file 1: Table S2.

**Fig. 5:**
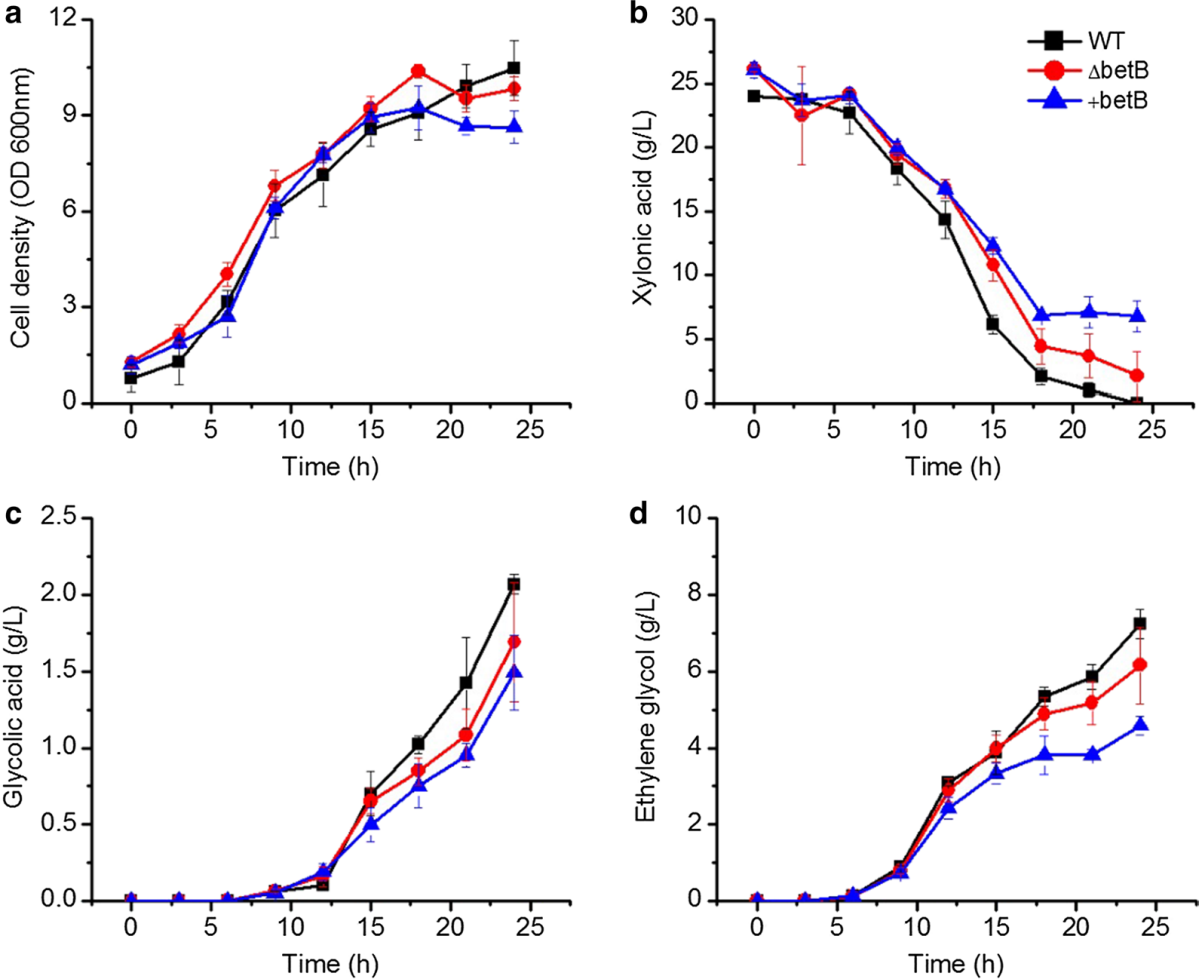
Growth and metabolite production of *E. cloacae* S1, *E. cloacae* Δ*betB* and *E. cloacae *+ *betB* grown on xylonic acid in shake flask batch culture. *E. cloacae* S1 (filled square), *E. cloacae* Δ*betB* (filled circle) and *E. cloacae *+ *betB* (filled triangle). Data points are the average of n = 3; error bars represent standard error

Glycolaldehyde dehydrogenase activity of cell lysate of *E. cloacae* S1 using NAD as cofactor was 0.0021 U/mgP. While no activity was measured using NADP as the cofactor. Among the purified enzymes, only BetB showed a distinct glycolaldehyde dehydrogenase activity of 0.21 U/mgP when using NAD as the cofactor. All other enzymes exhibited a very low level of glycolaldehyde dehydrogenase activity using NAD as the cofactor. When using NADP as the cofactor, all these selected enzymes showed a very low level of activity. These results indicate that BetB might be responsible for glycolic acid formation from glycolaldehyde. To further investigate the role of BetB in the glycolic acid formation from glycolaldehyde, a gene knock-out strain *E. cloacae* Δ*betB* and an over-expression strain *E. cloacae *+ *betB* were constructed. These strains were cultured in flasks for ethylene glycol production, and fermentation results are shown in Fig. 5.

The cell growth of these three strains was similar. Glycolic acid and ethylene glycol synthesized by *E. cloacae* Δ*betB* were 1.7 g/L and 6.2 g/L respectively, which were slightly decreased compared with that of the wild-type strain, the latter synthesized 2.1 g/L of glycolic acid and 7.2 g/L of ethylene glycol. However, glycolic acid and ethylene glycol synthesized by *E. cloacae *+ *betB* were 1.5 g/L and 4.6 g/L, thus slightly decreased compared with levels of wild type strain and *E. cloacae* Δ*betB*.

### Culture parameters optimization

**Fig. 6:**
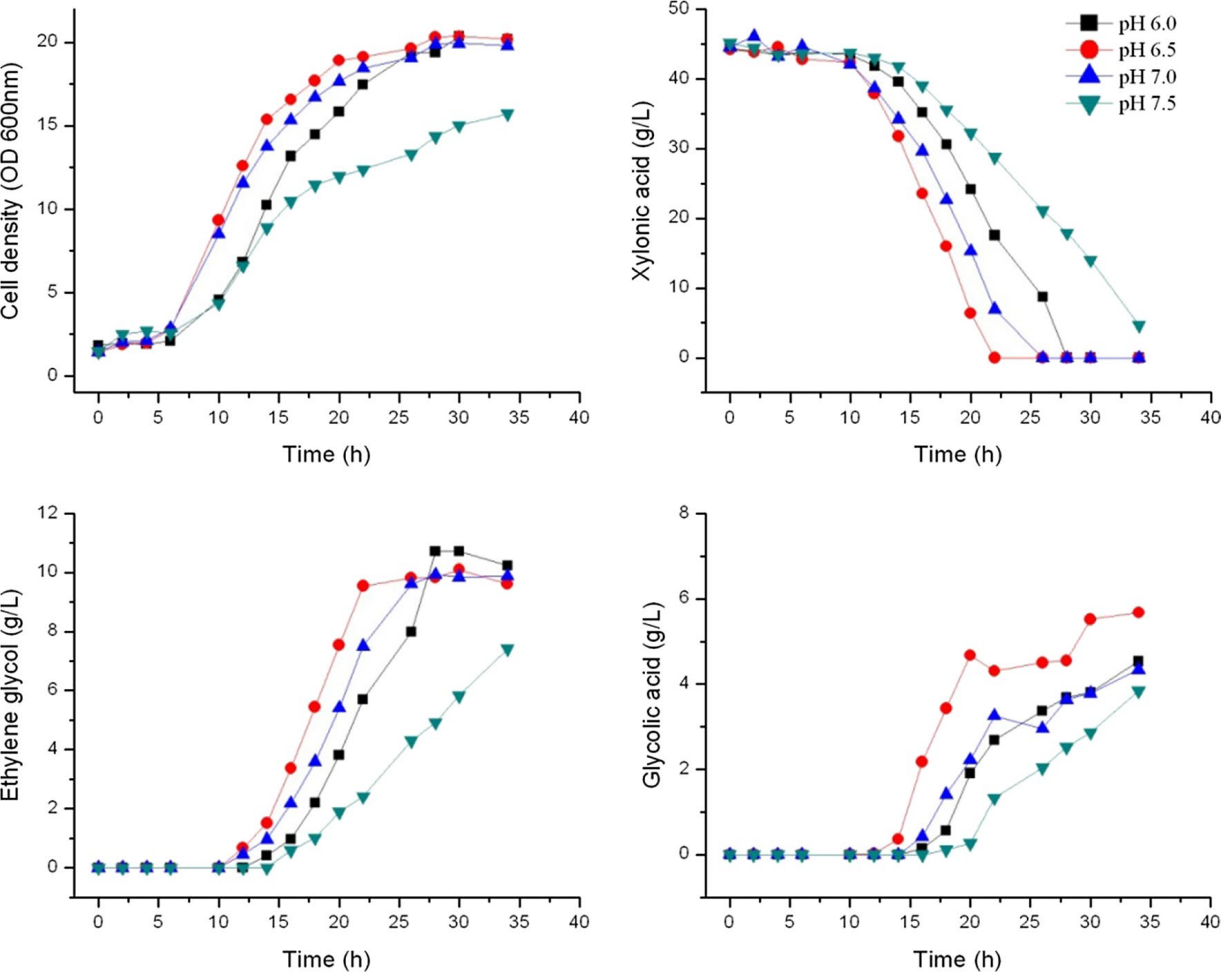
Cell growth and metabolite production of *E. cloacae* S1 grown on fermentation medium with xylonic acid in batch culture at different pH values in 5L bioreactors operated at 500 rpm

*E. cloacae* S1 was batch cultured in 5L stirred tank bioreactors for ethylene glycol and glycolic acid production. The culture pH was controlled at 6.0, 6.5 7.0 and 7.5, respectively. Agitation rate was maintained at 500 rpm, and cell growth and metabolites produced are presented in Fig. 6.

After 6 h of lag phase, cells started to grow and reached the exponential phase after about 10–12 h. Xylonic acid was not used by cells until cell density reached about OD 7. Cells could grow in the whole experimental culture pH range with cells at pH 6.5 had the fastest growth rate, whereas cells grown at pH 7.5 had the lowest growth rate. The effect of culture pH on cell growth, xylonic acid consumption, ethylene glycol, and glycolic acid production showed a similar trend with the pH 6.5 culture showing fastest utilization of xylonic acid in parallel with the fastest production of ethylene glycol and glycolic acid. Thus pH 6.5 was selected as the optimal culture pH.

**Fig. 7:**
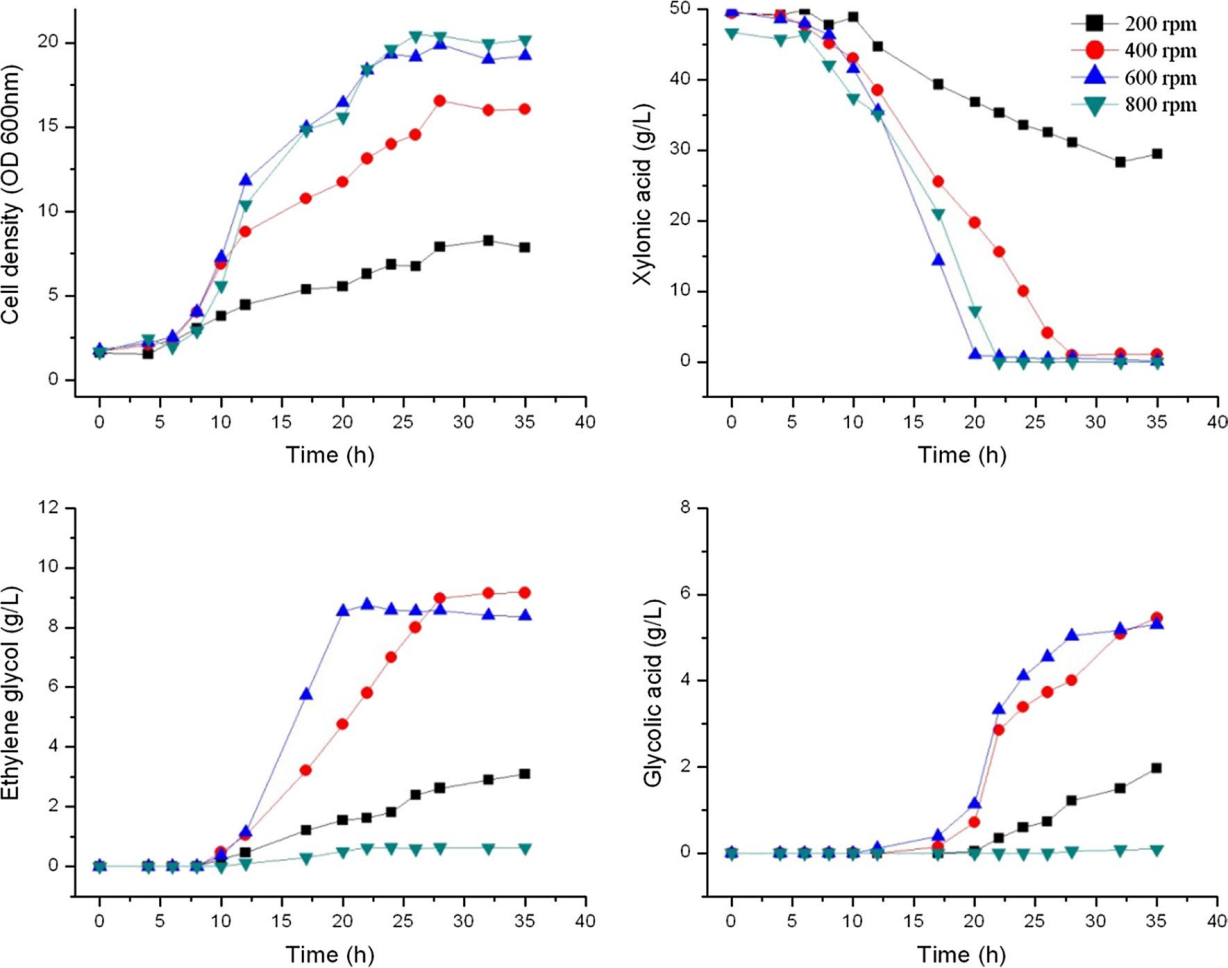
Cell growth and metabolite production of *E. cloacae* S1 grown on fermentation medium with xylonic acid in batch culture at different agitation rate in 5 L bioreactors operated at pH 6.5

Oxygen supplementation is a key parameter for cell growth and product synthesis. The agitation rate was set at 200, 400, 600 and 800 rpm to give micro-aerobic condition at the lowest rate to fully aerobic conditions at the highest rate, and culture pH was kept constant at pH 6.5. Fermentation results of *E. cloacae* S1 at different agitation rates are presented in Fig. 7.

Cells growth showed a positive correlation with agitation rate with cells grown at 600 rpm and 800 rpm gave the highest cell densities (OD 19.9 and 20.4 respectively), and those at 200 rpm had the lowest cell density (OD 8.0). The trend of xylonic acid consumption was similar to that of cell growth, with cells grown at 600 rpm gave the fastest xylonic acid consumption rate (3.8 g/Lh), and those grown at 200 rpm had the lowest xylonic acid consumption rate (0.9 g/Lh). Ethylene glycol and glycolic acid production were positively correlated to agitation rate from 200 to 600 rpm. However, the product synthesis was strictly inhibited at the condition of 800 rpm agitation. Thus, medium agitation rate appears to favor both ethylene glycol and glycolic acid synthesis, and therefore 600 rpm was selected as the optimal agitation condition.

### Ethylene glycol production in fed-batch fermentation

**Fig. 8:**
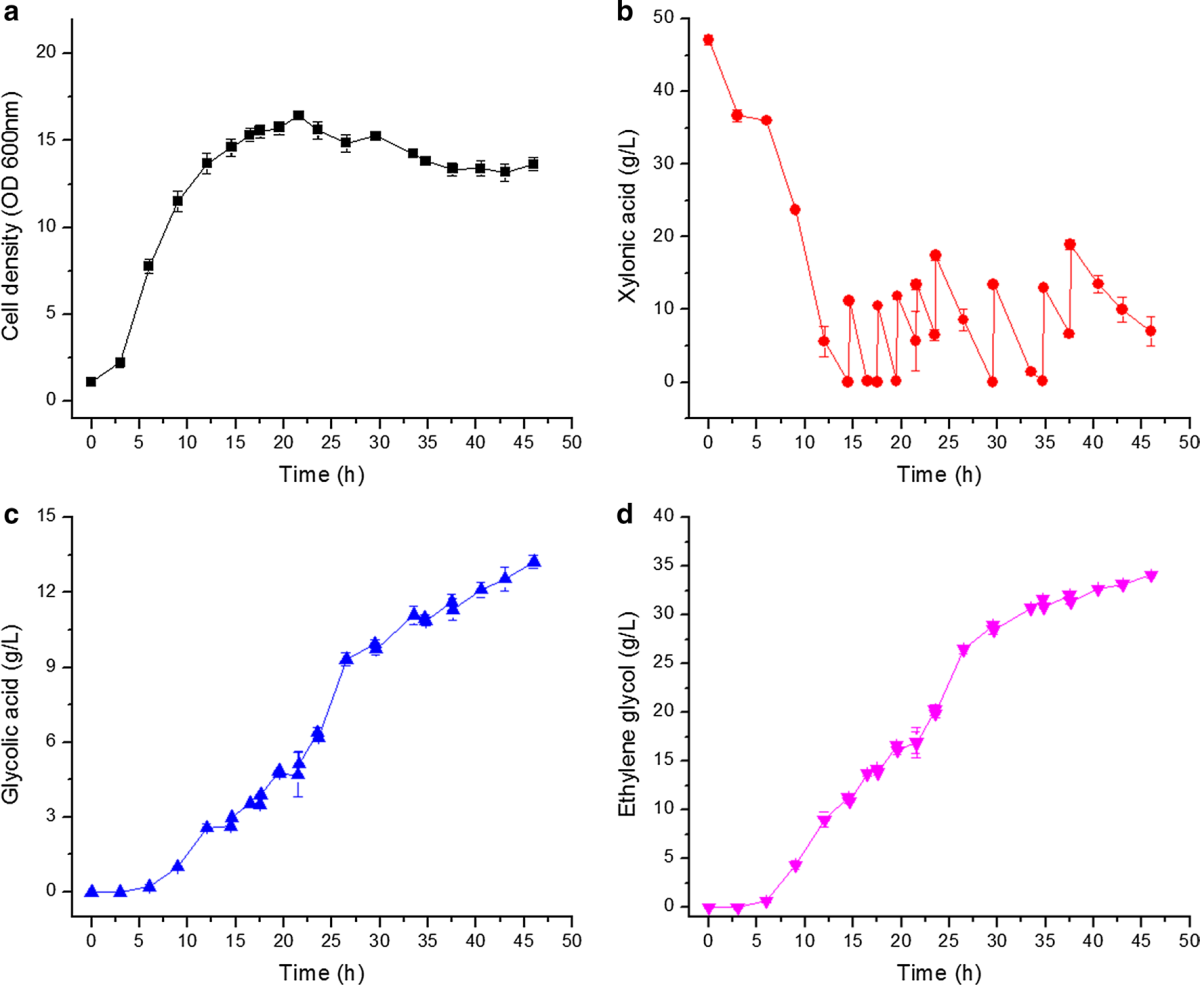
Cell growth and metabolite production of *E. cloacae* S1 grown on fermentation medium with xylonic acid in fed-batch culture at pH 6.5 values in a 5L bioreactor operated at 600 rpm. **a** Cell density; **b** xylonic aid; **c** glycolic acid; **d** ethylene glycol. Data points are the average of n = 3; error bars represent standard error

*Enterobacter cloacae* S1 was cultured in a 5 L stirred tank bioreactor, and xylonic acid was fed in the process using bolus additions. Fermentation results are presented in Fig. 8.

Similar to the batch fermentations, xylonic acid was quickly consumed after cells reached the exponential phase. After 15 h of batch culture, xylonic acid was fed for the first time, and 8 bolus additions of xylonic acid were made in total as shown in Fig. 8b. The highest cell density of 16.4 (OD) was reached at 21 h; after that cell density started to decrease. Ethylene glycol had a high production rate of 1.2 g/Lh from about 10 h to 30 h, and then the productivity decreased. The trend of glycolic acid synthesis was similar to that of ethylene glycol production. In total, 34.1 g/L ethylene glycol and 13.2 g/L glycolic acid were produced after 46 h of cultivation. The molecular conversion ratio calculated was 0.217 mol/mol for glycolic acid and 0.772 mol/mol for ethylene glycol, and the total conversion ratio reached 0.989 mol/mol xylonic acid.

## Discussion

### Xylonic acid utilization by microorganisms

Xylose is the second most abundant sugar in nature after glucose, and many microorganisms can catabolize xylose through the pentose phosphate pathway. However, catabolism of xylonic acid is not common by microorganisms. There are two pathways of xylonic acid catabolism have been reported in *Pseudomonas fragi*. One way consists of: d-xylose → d-xylonate → 3-deoxy-d-pentulosonic acid → α-ketoglutarate semi-aldehyde → α-ketoglutarate. α-Ketoglutarate is then fed into the TCA cycle for further metabolism. This pathway was named the Weimberg pathway, in recognition of the scientist Ralph Weimberg [13]. Another way contains the following steps: d-xylose → d-xylonate → 3-deoxy-d-pentulosonic acid → pyruvate + glycolaldehyde. This metabolic pathway was named the Dahms pathway after the scientist A. Stephen Dahms [14]. Glycolaldehyde produced can be converted to ethylene glycol by a reduction reaction or converted to glycolic acid by an oxidization reaction. Microorganisms that contain any of the two pathways can grow on xylonic acid as the sole carbon source. Our results showed that strains 2 and 3 could use xylonic acid as the sole carbon source, but no known metabolites were detected. Thus these two strains might contain the Weimberg pathway. Xylonic acid was used by strain 1 and *E. coli* W3110 and both ethylene glycol and glycolic acid were produced by these strains suggesting that these two strains might use the Dahms pathway. By contrast strain 4 doesn’t seem to possess any of the two pathways. It might use other bacteria’s metabolites as carbon source to grow in the enrichment medium and pass the enrichment process.

### Physiological characterization of *E. cloacae* S1

*Enterobacter cloacae* is a facultative anaerobic Gram-negative bacterium belonging to the family of *Enterobacteriaceae*. Like most *Enterobacter*, *E. cloacae* occurs as a commensal microorganism in water, sewage, soil, meat, hospital environments, the skin, and in the intestinal tracts of humans and animals [15]. In biotechnology, this bacterium was used as a producer of hydrogen and 2,3-butanediol, and the two chemicals were produced under anaerobic and aerobic conditions, respectively [16, 17]. Acetoin is an intermediate of 2,3-butanediol, and commonly produced together with 2,3-butanediol [18]. In this study, acetoin and 2,3-butanediol were the main metabolites of *E. cloacae* S1 using glucose, xylose, gluconic acid, and glycerol as carbon sources.

Xylonic acid is not a common chemical, and we have not found any reports about using xylonic acid as the sole carbon source for cultivation of microorganisms. Strains that can use xylonic acid as a carbon source must have the Weimberg pathway or Dahms pathway. Use of xylonic acid as a carbon source for microorganism culture increases the resistance to contamination. Xylonic acid used throughout this study was synthesized by *K. pneumoniae*, in which a PQQ-dependent glucose dehydrogenase catalyzed the reaction [7]. We have checked the genome of *E. cloacae* S1 and found that this bacterium has the gene coding for a PQQ-dependent glucose dehydrogenase, however, this bacterium does not hold the PQQ synthesis genes. Therefore, xylose cannot be converted to xylonic acid and was not further converted to ethylene glycol and glycolic acid by *E. cloacae*.

### Red recombinase associated gene recombination method is effective for *E. cloacae*

Gene recombination is a commonly used tool in molecular biology. Traditionally, suicide plasmid homologous recombination was used for gene recombination in bacteria, and it was used in *E. cloacae* until recently [17]. Red recombinase associated gene recombination was first developed in *E. coli* [19] and improved in *Streptomyces*. This method has the advantage of high efficiency and is easy to operate. Linear DNA with 36-nt homologous extensions was sufficient to obtain successful recombination [12]. The Red recombinase system has been modified as recombination tool suitable for many microorganisms, such as *Burkholderia cepacia* [20] *Pseudomonas aeruginosa* [21], *Pantoea ananatis* [22], *Salmonella enterica* [23], and *Vibrio cholerae* [24]. However, the minimal sizes of homologous extension are different ranging from 50 to 1000-nt. Initially no colony was obtained on selection plates using liner DNA with 39 and 40 nt homologous extensions in this study. Linear DNA with long homologous extensions was constructed following the method we have developed for gene recombination in *K. pneumonia*, of which high recombination ratio was obtained with linear DNA containing 500 nt homologous extensions [11]. Similarly, high recombination ratio was obtained in *E. cloacae* in this study with 500 nt homologous extensions, and successful recombinants was obtained after a single experiment.

### The function of genes in the *yjh* operon

*Enterobacter cloacae* Δ*yjhG* and *E. cloacae* Δ*yjhH* can grow with xylose as the sole carbon source, but not with xylonic acid (Fig. 2). It indicated that YjhG and YjhH were responsible for glycolaldehyde synthesis from xylonic acid, and these two enzymes have no isoenzymes in *E. cloacae*. This finding is different to *E. coli*, where the two enzymes both have an isoenzyme [2]. Genes in *yjh* operon were suspected to be important for xylose or xylonic acid metabolism, since *yagG* has been noted as a putative d-xylonate transporter for xylonic acid catabolism in *E. coli* [25]. However, the xylose metabolism was not affected by disrupting any of these genes (Figs. 2, 3). Thus it appears that *yjh* operon is not directly involved in xylose metabolism. Excluding *yjhG* and *yjhH*, the activities of other genes in *yjh* operon have no effect on xylonic acid catabolism. Further work is needed to determine the native physiological function of this operon.

### Identification of genes responsible for ethylene glycol and glycolic acid synthesis from glycolaldehyde

The *E. cloacae* YqhD has ethylene glycol dehydrogenase activity, similar to the YqhD in *E. coli* [2]. However, this enzyme was not solely responsible for this reaction. Generally, many short-chain alcohol dehydrogenases have a broad substrate range. Other short-chain alcohol dehydrogenases in the cell might be responsible for ethylene glycol synthesis in *E. cloacae* ΔyqhD.

Some strains of *E. cloacae* have homologous genes of *aldA*, however, some strains including *E. cloacae* S1 do not have this gene in their genome. *betB* encodes a betaine aldehyde dehydrogenase. The substrate specificity of this enzyme from *E. coli* was strictly limited to betaine aldehyde [26]. By contrast our results showed that this enzyme catalyzes the reaction of glycolaldehyde oxidation to glycolic acid in vitro. However, the in vivo experimental results show that this enzyme was not the enzyme responsible for glycolic acid formation from glycolaldehyde. Further research is needed to identify the enzyme that responsible for this reaction.

### Ethylene glycol and glycolic acid synthesis have an inherent relationship

YqhD was responsible for ethylene glycol synthesis from glycolaldehyde, and ethylene glycol synthesis was reduced in *E. cloacae* Δ*yqhD*. As glycolaldehyde synthesis was not being affected in *E. cloacae* Δ*yqhD*, we hypothesized that glycolic acid synthesis would not be affected. However, glycolic acid synthesis was also decreased (Fig. 4). Furthermore, ethylene glycol and glycolic acid synthesis did not change in the *yqhD* over-expression strain. Similarly, glycolic acid synthesis was decreased in *E. cloacae* ΔbetB and *E. cloacae *+ *betB*, and ethylene glycol synthesis was also decreased (Fig. 5). This finding is different from the metabolite production of engineered *E. coli*, in which over-expression of *yqhD* resulted in an increase of ethylene glycol but a decrease of glycolic acid synthesis [2]. In the culture parameter optimization experiments, ethylene glycol production varied in different conditions. Glycolic acid produced in these experiments showed a similar trend to that of ethylene glycol (Fig. 6, 7). Thus, the formation of ethylene glycol and glycolic acid are closely linked in *E. cloacae*. This is in contrast to production of these two metabolites in engineered *E. coli* in which fully aerobic condition favor ethylene glycol formation and microaerobic condition favor glycolic acid formation [3]. The mechanism of this relationship needs further investigation.

### Ethylene glycol production by *E. cloacae*

Different ethylene glycol synthesis pathways have been constructed, and several bacteria have been used as host cells. Utilising the Dahms pathway, 11.7 g/L ethylene glycol was produced from 40 g/L xylose by an engineered *E. coli* strain, with the productivity of 0.24 g/L h [2]. Furthermore, 20 g/L of ethylene glycol was produced with a molar yield of 0.38 g/g xylose and productivity of 0.37 g/L h by a modified strain of *E. coli* using xylulose as an intermediate [3]. In another study 40 g/L ethylene glycol was produced with a yield of 0.63 g/g xylose and productivity of 0.55 g/L h after some optimization of the conditions [4]. Using glucose as substrate, 3.5 g/L ethylene glycol was produced by engineering *C. glutamicum*, with a yield of 0.08 g/g glucose and productivity of 0.05 g/L h [5]. Using *E. coli* as the host cell, 4.1 g/L ethylene glycol was produced with a yield of 0.14 g/g glucose and productivity of 0.03 g/L h were obtained [6]. In this report, 34.1 g/L ethylene glycol was produced, with the yield 0.288 g/g xylonic acid and maximum productivity of 0.74 g/L h. The productivity obtained here is higher than these previous published reports that using xylose or glucose as the substrate. Based on the amount of xylonic acid supplied, the total molecular conversion ratio reached nearly 1 mol/mol xylonic acid. The high conversion ratio indicates that all the xylonic acid added was metabolized in the cell through one pathway, and the glycolaldehyde formed was completely converted to ethylene glycol and glycolic acid. However, pyruvate produced in the process was utilized by cells. In a research that using engineered *E. coli* for ethylene glycol and glycolic acid production, pyruvate was partly recovered for glycolic acid synthesis and the total yield of the process was improved [4]. The ability of convert xylonic acid to ethylene glycol by *E. cloacae* S1 was better than that of *E. coli* W3110 (Table 1). Thus, *E. cloacae* S1 might be a better chassis for further metabolic engineering to improve ethylene glycol and glycolic acid production. Recently, there are two reports of ethylene glycol production that both achieved very high final product levels [27, 28]. They have a common characteristic that the reaction of xylose flowing into the pentose phosphate pathway was kept active. This is different to all other reports of using xylose as carbon source for ethylene glycol production, where the pentose phosphate pathway was inactivated to prevent flow of xylose into it. In one of the recent reports, *yqhD* was replaced by *fucO*, coding for a NADH dependent dehydrogenase, leading to a distinct increase in ethylene glycol titer of > 70 g/L [27]. While the engineered *E. coli* strain in the other report used *yqhD*, and with precise control of key genes expression resulting to even higher product titers of 108 g/L [28]. Adopting these metabolic engineering strategies to modify *E. cloacae* S1, ethylene glycol and glycolic acid production might be further improved.

Ethylene glycol and glycolic acid synthesis by *E. cloacae* started after ~ 10 h of cultivation, and entered a high rate after around 12 h. However, cell growth rate was highest between 3–12 h (Figs. 4, 5, 6, 7, 8). Thus cell growth and the synthesis of ethylene glycol and glycolic acid were not coincided. This is different to all reports that using *E. coli* as the producer, in which the cell growth and ethylene glycol synthesis are coincided [3, 4]. Ethylene glycol and glycolic acid syntheses were inhibited at an agitation rate of 800 rpm, but xylonic acid consumption proceeded at a high rate (Fig. 7). This indicates that some other metabolites were generated in the process, which is interesting for further investigation.

## Conclusions

Ethylene glycol is a highly important commodity chemical. However, there are no known natural pathways to directly synthesize ethylene glycol from carbohydrates [29, 30]. In this study, it was shown that ethylene glycol can be produced by *E. cloacae* S1 using xylonic acid as the sole carbon source. This synthesis pathway presents an alternative route for ethylene glycol production from sugars. Ethylene glycol production by *E. cloacae* S1, a native producer, has a high productivity and titer. This was achieved with little process optimisation and it is anticipated that the fed-batch process can be further improved in terms of product titer and yield. This work forms the basis to develop a new industrial process for ethylene glycol and glycolic acid production by a biological route.

## Methods

### Strains, plasmids, and primers

Bacterial strains and plasmids used in this study are listed in Table 2. Primers used for PCR are listed in Additional file 1: Table S3.

**Table 2:** Strains and plasmids

Strain or plasmid	Relevant genotype and description	Reference or source
*K. pneumoniae* Δ*gad*	Δ*gad*	[7]
*E. coli* W3110	Wild type	Lab stock
*E. coli* BL21/yqhD	Over-expression of *yqhD*	This work
*E. coli* BL21/aldB	Over-expression of *aldB*	This work
*E. coli* BL21/betB	Over-expression of *betB*	This work
*E. coli* BL21/ad1	Over-expression of *ad1*	This work
*E. coli* BL21/ad2	Over-expression of *ad2*	This work
*Enterobacter cloacae* S1	Wild type,	This work
*E. cloacae* Δ*yjhG*	Δ*yjhG*, Str^r^	This work
*E. cloacae* Δ*yjhH*	Δ*yjhH*, Apr^r^	This work
*E. cloacae* Δ*yhcH*	Δ*yhcH*, Apr^r^	This work
*E. cloacae* Δ*yagG*	Δ*yagG*, Apr^r^	This work
*E. cloacae* Δ*iclR*	Δ*iclR*, Apr^r^	This work
*E. cloacae* Δ*xyL*	Δ*xyl*, Apr^r^	This work
*E. cloacae* Δ*yqhD*	Δ*yqhD*, Str^r^	This work
*E. cloacae* Δ*betB*	Δ*betB*, Str^r^	This work
*E. cloacae* +*yqhD*	pSARI-yqhD, Kan^r^	This work
*E. cloacae* +*betB*	pSARI-betB, Kan^r^	This work
pMD18-T-simple	Amp^r^, TA cloning vector, 2692 bp	Takara^®^
pMD18T-yhcH	Amp^r^, carries *yhcH*, 4,237 bp	This work
pMD18T-ΔyhcH	Amp^r^, carries part of *yhcH*, Apr^r^, 5077 bp	This work
pMD18T-yjhH	Amp^r^, carries *yjhH*, 4,844 bp	This work
pMD18T-ΔyjhH	Amp^r^, carries part of *yjhH*, Apr^r^, 5285 bp	This work
pMD18T-yjhG	Amp^r^, carries *yjhG*, 6034 bp	This work
pMD18T-ΔyjhG	Amp^r^, carries part of *yjhG*, Str^r^, 5416 bp	This work
pMD18T-yagG	Amp^r^, carries *yagG* gene, 5237 bp	This work
pMD18T-ΔyagG	Amp^r^, carries part of *yagG* gene, Apr^r^, 5897 bp	This work
pMD18T-xyL	Amp^r^, carries β-xylosidase gene, 5642 bp	This work
pMD18T-ΔxyL	Amp^r^, carries part of β-xylosidase gene, Apr^r^, 6187 bp	This work
pMD18T-iclR	Amp^r^, carries *iclR*, 4637 bp	This work
pMD18T-ΔiclR	Amp^r^, carries part of *iclR*, Apr^r^, 5228 bp	This work
pMD18T-yqhD	Amp^r^, carries *yqhD*, 3856 bp	This work
pMD18T-ΔyqhD	Amp^r^, carries part of *yqhD*, Str^r^, 4268 bp	This work
pMD18T-betB	Amp^r^, carries *betB*, 5308 bp	This work
pMD18T-ΔbetB	Amp^r^, carries part of *betB*, Str^r^, 5261 bp	This work
pIJ773	Apr^r^, *aac(3)IV* with FRT sites, 4334 bp	[12]
pIJ778	Str^r^, *aadA* with FRT sites, 4337 bp	[12]
pIJ790	Cm^r^, encodes λ-Red genes, 6084 bp	[12]
pSARI	Kan^r^, PR, 4,914 bp (Genbank MH037013)	Lab stock
pSARI-red	Kan^r^, carries λ-Red genes, 6,799 bp	This work
pSARI-yqhD	Kan^r^, carries the *yqhD*, 6078 bp	This work
pSARI-betB	Kan^r^, carries the *betB*, 6630 bp	This work
Pet 28a	Vector carries N-terminal His Tag, Kanr, 5369 bp	Novagen^®^
Pet 28a-yqhD	Amp^r^, carries the *yqhD*, 6520 bp	This work
Pet 28a-aldB	Kan^r^, carries the *aldB*, 6895 bp	This work
Pet 28a-betB	Kan^r^, carries the *betB*, 6772 bp	This work
Pet 28a-ad1	Kan^r^, carries the *ad1*, 6727 bp	This work
Pet 28a-ad2	Kan^r^, carries the *ad2*, 6826 bp	This work

### Xylonic acid preparation

Xylonic acid (Ammonium salt) was produced from xylose by *K. pneumoniae*, as described previously [7]. The fermentation broth was centrifuged to eliminate cells and other insoluble impurities. 1% of activated carbon was added to the supernatant and filtrated with paper. The discolored liquid was concentrated to 700 g/L with a rotary evaporator at 70 °C. The xylonic acid crystals were formed after keeping the liquid at room temperature for 1 week. This xylonic acid obtained was used in the following experiments.

### Microorganisms screening and identification

Soil samples were collected from the campus of Shanghai Advanced Research Institute. 1 g of soil sample was inoculated to a 250 ml flask with 50 ml enrichment medium and then incubated aerobically at 37 °C on a rotary shaker (120 rpm). After one day of incubation, 0.1 ml of the culture broth was transferred to another flask with the same enrichment medium and incubated for 1 day. The enrichment medium used was M9 medium containing 40 g/L xylonic acid. After 3 rounds of such enrichment operation, 1 ml of 10^8^-fold diluted culture broth was plated on Luria–Bertani (LB) agar plate and cultured at 37 °C overnight. Colonies grown on the plates were inoculated to a 250 ml flask with 50 ml confirmation medium and then incubated on a rotary shaker at 37 °C and 120 rpm for 1 day. The confirmation medium contained: xylonic acid 40 g/L, Yeast extract 5 g/L, Tryptone 10 g/L, NaCl 10 g/L. Chemical compounds in the broth including xylonic acid and metabolites were quantified by high performance liquid chromatography (HPLC) as described previously [7].

16S rRNA gene of the selected strain was sequenced. The 16S rRNA gene sequence was blasted in the NCBI, and a dendrogram was composed to elucidate evolutionary relationships between selected strain and related strains. This analysis was used for strains identification.

### Flasks culture and medium

Wild-type and constructed *E. cloacae* strains were inoculated in 250 ml flasks containing 50 ml medium and incubated on a rotary shaker at 37 °C and 120 rpm for 1 day. All experiments were done in triplicate, and data are expressed as the mean ± standard error.

M9 medium with glucose, gluconic acid, 2-ketogluconic acid, xylose, xylonic acid or glycerol as the sole carbon source was used. If not mentioned, the concentration of the carbon source was 20 g/L. Gluconic acid and 2-ketogluconic acid used were in the form of sodium salt, and 2-ketogluconic acid was prepared as reported previously [31].

The fermentation medium contained: xylonic acid 30 g/L, corn steep liquor 4 g/L, (NH_4_)_2_SO_4_ 5 g/L, KCl 0.4 g/L, and MgSO_4_ 0.1 g/L.

### Ethylene glycol and glycolic acid structure confirmation

Glycolic acid produced by *E. cloacae* S1 was purified from the fermentation broth by ion-exchange chromatography and the structure was confirmed by nuclear magnetic resonance (NMR) spectroscopic analysis. A Bruker spectrometer was used and chemical shift values were reported in ppm (δ).

Ethylene glycol was confirmed by comparison with the standard chemical by HPLC [7] and gas chromatography (GC). A gas chromatograph system (Shimadzu GC 2010) equipped with a flame ionization detector and a DB-WAX column (30 m × 0.25 mm), with nitrogen as the carrier gas was used.

### Construction of mutants of *E. cloacae*

For mutant constructions, *E. cloacae* and *E. coli* were grown in Luria–Bertani (LB) medium at 37 °C. The antibiotics used in the selective medium were ampicillin (50 μg/mL), kanamycin (50 μg/mL), apramycin (50 μg/mL), and streptomycin (25 μg/mL). Red recombinase encoding genes were amplified from PIJ790 and ligated into pSARI to generate plasmid pSARI-red. This plasmid was transferred into *E. cloacae* to obtain *E. cloacae*/red.

*Enterobacter cloacae* Δ*yjhG* construction is described in detail as an example. Other mutants were constructed in the same way using corresponding primers and resistance genes.

The *yjhG* gene in the genome of *E. cloacae* and flanking sequences was amplified by PCR using the primer pair yjhG-s and yjhG-a. The PCR product was ligated into the pMD18-T-simple vector to generate pMD18-T-yjhG. A linear DNA with 39 and 40 nt homologous extensions flanking streptomycin resistance gene *aadA* was amplified with plasmid pIJ778 as the template using the primer pair yjhG-FRT-s/yjhG-FRT-a. pMD18-T-ΔyjhG was constructed by replacing *yjhG* in plasmid pMD18-T-yjhG with the *aadA* cassette using the Red recombination system in *E. coli*.

The plasmid pMD18-T-ΔyjhG was further used as the template for PCR preparation of a linear DNA containing the streptomycin resistance gene *aadA* with 500 bp of homologous regions on both sides. Finally, the linear DNA was transformed into *E. cloacae*/red, which already hosted the plasmid pSARI-red. Homologous recombination between the linear DNA and the chromosome was facilitated by Red recombinase and led to *yjhG* deletion in *E. cloacae*.

### Construction of strains for protein over-expression

The ORF of *yqhD* in *E. cloacae* S1 was amplified using the primer pair yqhD-s2 and yqhD-a2. The PCR product was ligated into the pMD18-T-simple vector to generate pMD18-T-yqhD. The latter was digested with BamH I and Nco I to obtain the *yqhD* fragment, and this fragment was ligated into pET28a to generate pET28a-yqhD. pET28a-yqhD was transformed into *E. coli* BL21 for protein expression. *E. coli* BL21/aldB, *E. coli* BL21/betB, *E. coli* BL21/ad1, and *E. coli* BL21/ad2 were constructed in the same way as *E. coli* BL21/yqhD.

pMD18T-yqhD was digested and ligated into pSARI to generate SARI-yqhD. SARI-yqhD was transformed into *E. cloacae* S1 to obtain *E. cloacae *+ *yqhD. E. cloacae *+ *betB* was constructed following the same method.

### Enzyme preparations and assay

YqhD and other enzymes were purified from the lysate of *E. coli* BL21/yqhD and other *E. coli* strains by affinity chromatography using a His-Trap column. The enzyme assay follows the method for 2,3-butanediol dehydrogenase activity assay [18]. Ethylene glycol or glycolaldehyde was used as substrates.

### Culture parameters optimization and fed batch culture condition

Stirred tank bioreactors were used for culture parameters optimization. For the seed culture, 250-mL flasks containing 50 mL of LB medium were incubated on a rotary shaker at 37 °C and 200 rpm overnight. The seed culture was inoculated into a 5-L bioreactor (BIOSTAT-A plus Sartorius) with a working volume of 3 L and air flow rate of 2 L/min. Culture pH and stirring rate were optimized individually.

Fed batch cultures were performed at optimized conditions, with culture pH 6.5, culture temperature 37 °C and agitation rate of 600 rpm. When xylonic acid in the broth was consumed to 5 g/L, 100 ml 500 g/L of xylonic acid solution was added. All experiments were done in triplicate, and data were expressed as the mean ± standard error.

## Supplementary information

**Additional file 1.** Additional tables and figures.

## Data Availability

The 16S rRNA gene sequence has been submitted to GenBank with the accession number of MG779638. The genome sequence data was submitted to submitted to GenBank with the accession number of VSZU00000000.
