# Supplementary material for: Ethylene glycol and glycolic acid production from xylonic acid by Enterobacter cloacae
Source: Microb Cell Fact. 2020 Apr 15;19:89. doi: 10.1186/s12934-020-01347-8 (PMC7158088; doi:10.1186/s12934-020-01347-8)
Supplement: Supplementary file 1 — Additional file 1. Additional tables and figures. [file 12934_2020_1347_MOESM1_ESM.docx]

Additional materials

Table S1. Metabolites of *E. cloacae* S1 using different carbon sources. Data shown are averages of triplicate experiments with standard error.

| Carbon source | Metabolites (g/L) | | | | |
| --- | --- | --- | --- | --- | --- |
|  | Ethylene glycol | Glycolic acid | Acetic acid | Acetoin | 2,3-butanediol |
| Xylonic acid | 2.67±0.26 | 0.46±0.08 | 0 | 0 | 0 |
| Xylose | 0 | 0 | 0 | 0 | 1.18±0.13 |
| Glucose | 0 | 0 | 1.15±0.02 | 0.05±0.05 | 2.14±0.18 |
| Gluconic acid | 0 | 0 | 0.51±0.43 | 1.49±0.05 | 1.56±0.05 |
| 2-Ketogluconic acid | 0 | 0 | 1.76±0.21 |  |  |
| Glycerol | 0 | 0 | 0. | 0.77±0.38 | 0.45±0.15 |

Table S2. Enzyme activity determination of purified putative glycolaldehyde DH expressed in *E. coli* (AlB, BetB, Ad1 and Ad2) compared with cell lysate of *E. cloacae* S1.

| Cofactor used | Glycolaldehyde dehydrogenase activity (U/mg P) | | | | |
| --- | --- | --- | --- | --- | --- |
|  | Cell lysate | AldB | BetB | Ad1 | Ad2 |
| NAD | 0.0021± 0.0004 | 0.025±0.008 | 0.21± 0.12 | 0.024± 0.02 | 0.012± 0.005 |
| NADP | 0±0.01 | 0.035±0.015 | 0.042±0.003 | 0.025±0.02 | 0.003±0.003 |

Table S3 Primers

| Primer name | Sequence (5’-3’) | |
| --- | --- | --- |
| 16sRNA-a | GGGAGCGAATCAGACGACG | |
| 16sRNA-s | GGTTACCTTGTTACGACTT | |
| yhcH-a | | GCTTCAGACACCTTCCAGTAGTAGGGGTTG |
| yhcH-s | | TCATTTGCATGATTCGCATCAGATAATGATTA |
| yjhH-a | GGGAGCGAATCAGACGACG | |
| yjhH-s | TCGGGCAAACACGACATCA | |
| yjhG-a | GTCAGCAGAATGTAGGTTACGCAG | |
| yjhG-s | ACCCCTACTACTGGAAGGTGTCTG | |
| yagG-a | | GCCCCCACGGACGATAGAG |
| yagG-s | | CTGCGCGACAACGACATCA |
| xyL-a | | CGGAGAACGGCAAGTATCG |
| xyL-s | | ATCGTCGCCTACTCGCTGA |
| iclR-a | | GATGGGCGAAGCCAAAGAG |
| iclR-s | | GCTCTACGGCAACGACTCG |
| yqhD-a | TTAGCGCGCCGCCTCGTAGATA | |
| yqhD-s | ATGAATAATTTTAATCTCCACACCCCAACCCGC | |
| betB-a | ATGCACCACATCGAACAGATTTT | |
| betB-s | CTTCCACCAGAAAATGCGCCTCAA | |
| yhcH-FRT-a | | TTACCCCACCAACATCTTAACAACCACTTTTCTGACCTTATTCCGGGGATCCGTCGACC |
| yhcH-FRT-s | | TTGAATTCTCGTATAAAAGATAAAAATGAAACGGTGTTTTTGTAGGCTGGAGCTGCTTC |
| yjhH-FRT-a | TCAGCAGAGCTTGAGCTGTTGCAGCAGGGTTTTCAGCTGATTCCGGGGATCCGTCGACC | |
| yjhH-FRT-s | ATGCCGCAGTCCGCGTTGTTCACGGGAATCATTCCCCCTGTGTAGGCTGGAGCTGCTTC | |
| yjhG-FRT-a | TTAAAGTCCGAGTGCTTTTTTACCGGCGTTAATTACCTCATTCCGGGGATCCGTCGACC | |
| yjhG-FRT-s | ATGACCATCGAGAAAATTTTCACCCCGCAGGATGATGCGTTGTAGGCTGGAGCTGCTTC | |
| yjhG-FRT-s | | ATGACCATCGAGAAAATTTTCACCCCGCAGGATGATGCGTTGTAGGCTGGAGCTGCTTC |
| yagG-FRT-a | | TTACTGGGATGCGGCTGTCGCGGCGTCGGGAAGGGCCTCATTCCGGGGATCCGTCGACC |
| xyL-FRT-a | | CACGCGCGCTGGGCTACCCGGCCTGAGCCGTTAAATCGGATTCCGGGGATCCGTCGACC |
| xyL-FRT-s | | ATGGAAATCACCAACCCAATACTCACCGGCTTCAACCCGGTGTAGGCTGGAGCTGCTTC |
| iclR- FRT-a | | ATGCCGATTATTCAGTCTGTTGAACGTGCGTTGCAGATCATTCCGGGGATCCGTCGACC |
| iclR- FRT-s | | TCAGGCCGGGTAGCCCAGCGCGCGTGAGAGCTGGAGCCCGTGTAGGCTGGAGCTGCTTC |
| yqhD- FRT-a | ATTTTGGCATCCACCGGATAAGTCACGTACTGCTCAACGATTCCGGGGATCCGTCGACC | |
| yqhD- FRT-s | TCCGGTGTACACCTACACCCTGCCCGCGCGTCAGGTCGCGTGTAGGCTGGAGCTGCTTC | |
| betB- FRT-a | TGCCGCATTGCCAGCGGGCGTGTTTAATATCATCACCGGGATTCCGGGGATCCGTCGACC | |
| betB- FRT-s | TTTTTGTAGGCCCGGTAAGCGTAGCGCCACCGGGCAGGGTTGTAGGCTGGAGCTGCTTCGAA | |
| Test773-s | ATGACGAGCGTCCCTCCC | |
| Test 778 | AGAATCTCGCTCTCTCCAGGGGAAG | |
| yqhD-a2 | CATGCCATGGTTAGCGCGCCGCCTCGTAGA | |
| yqhD-s2 | CGCGGATCCATGAATAATTTTAATCTCCACACCCCAACC | |
| aldB-a | | CCCAAGCTTGGGCTAGAACAGCCCCAGCGGT |
| aldB-s | | CCGGAATTCCGGATGACAAACAATCCTCCCTCTTCA |
| betB-a2 | | CCCAAGCTTGGGTTAACCCACAATCGACTTCGG |
| betB-s2 | | CGCGGATCCGCGATGCACCACATCGAACAGATTT |
| ad1-a | | CCGGAATTCCGGTTACTTACGCACCACCACCAGC |
| ad1-s | | CGCGGATCCGCGATGGCTTATCAGACAGTAAATCCTGC |
| ad2-a | CCGGAATTCCGGTCAGTCGCACCAGTGGTTGA | |
| ad2-s | CGCGGATCCGCGATGGACGATCTGAAGATATTTATCG | |

Figure S1 Phylogenetic tree of the16S rRNA genes of *E. cloacae* S1 and other *E. cloacae* strains

Figure S2. Ethylene glycol and glycolic acid identification. A: ^1^H NMR spectra of glycolic acid; B: ^13^C NMR spectra of glycolic acid; C: HPLC chromatograms of ethylene glycol; D GC chromatograms of ethylene glycol. Pink lines are standard chemicals, blue lines are samples.
